# Surgical Management for Dystonia: Efficacy of Deep Brain Stimulation in the Long Term

**DOI:** 10.3390/neurolint13030037

**Published:** 2021-08-02

**Authors:** Walaa A. Kamel, Pritam Majumdar, Georgios Matis, Albert J. Fenoy, Shankar Balakrishnan, Ali T. Zirh, Aslihan Cevik, Amit Kumar Tomar, Naoufel Ouerchefani

**Affiliations:** 1 Neurology Department, Faculty of Medicine, Beni-Suef University, Beni-Suef 62511, Egypt; walaaneuro@yahoo.com; 2 Neurology Department, Ibn-Sina Hospital, Kuwait City 25427, Kuwait; 3 Department of Stereotactic and Functional Neurosurgery, University Cologne Hospital, 50931 Cologne, Germany; georgios.matis@uk-koeln.de; 4 Department of Neurosurgery, McGovern Medical School, The University of Texas at Houston, UTHealth Neurosciences, Houston, TX 77030, USA; Albert.J.Fenoy@uth.tmc.edu; 5 Department of Neurology and Neuromodulation, MIOT International Hospital, Hennai 600089, India; dr.shankar.dm@gmail.com; 6 Department of Neurosurgery, Istanbul Medipol University, Istanbul 34810, Turkey; ali@alizirh.com; 7 Department of Neurosurgery, Istanbul Aydin University, Istanbul Medical Park Florya, Istanbul 34295, Turkey; aslihancevik@gmail.com; 8 Department of Anesthesia and Neurosurgery, Indo-Gulf Hospital, Noida 201301, India; dramittomar@gmail.com; 9 Department of Neurosurgery, Foch Hospital, 92150 Paris, France; n.ouerchefani@hopital-foch.com

**Keywords:** deep brain stimulation (DBS), Global Dystonia Severity scale (GDS), Burke–Fahn–Marsden dystonia rating scale (BFMDRS), subthalamic nucleus (STN), globus pallidus interna (GPi)

## Abstract

Introduction: Dystonia is a movement disorder substantially affecting the quality of life. Botulinum Neurotoxin (BoNT) is used intramuscularly as a treatment for dystonia; however, not all dystonia patients respond to this treatment. Deep brain stimulation (DBS) is an established treatment for Parkinson’s disease (PD) and essential tremor, but it can help in dystonia as well. Objectives: We studied a total of 67 dystonia patients who were treated with DBS over a period of 7 years to find out the long-term efficacy of DBS in those patients. First, we calculated patient improvement in post-surgery follow-up programs using the Global Dystonia Severity scale (GDS) and Burke–Fahn–Marsden dystonia rating scale (BFMDRS). Secondly, we analyzed the scales scores to see if there was any statistical significance. Methods: In our study we analyzed patients with ages from 38 to 78 years with dystonia who underwent DBS surgery between January 2014 and December 2020 in four different centers (India, Kuwait, Egypt, and Turkey). The motor response to DBS surgery was retrospectively measured for each patient during every follow-up visit using the GDS and the BFMDRS scales. Results: Five to 7 years post-DBS, the mean reduction in the GDS score was 30 ± 1.0 and for the BFMDRS score 26 ± 1.0. The longitudinal change in scores at 12 and 24 months post-op was also significant with mean reductions in GDS and BFMDRS scores of 68 ± 1.0 and 56 ± 1.0, respectively. The *p*-values were <0.05 for our post-DBS dystonia patients. Conclusions: This study illustrates DBS is an established, effective treatment option for patients with different dystonias, such as generalized, cervical, and various brain pathology-induced dystonias. Although symptoms are not completely eliminated, continuous improvements are noticed throughout the post-stimulation time frame.

## 1. Introduction

Dystonia is the third most common movement disorder after essential tremor and Parkinson’s disease (PD). Dystonia is normally considered a heterogeneous group of disorders characterized by abnormal muscle contractions leading to abnormal body posture [1]. Recently, the International Movement Disorders Society (MDS) proposed new classifications and approaches that can help to differentiate dystonia and its subgroups. According to MDS classifications, group I describes the clinical characteristics of the disease based on the patient’s age of onset, body distribution (focal, segmental, generalized), temporal pattern (persistent, action induced, paroxysmal), and associated with other features such combined or isolated symptoms. On the contrary, group II describes the etiology of the disorders such as inherited or acquired, and also with clinical evidence such as brain injuries or genetic causes [1,2]. These classifications help to avoid previous terming of primary or secondary dystonias where clinicians would provide an etiological diagnosis for patients that remain unclear despite extensive workups for new genetic etiologies [2]. In the past, clinicians were afraid of performing DBS surgeries on so-called secondary or “non-primary” dystonias because they believed that the success rate in these diagnoses was much lower. Currently, there are few medications available for treatment of dystonia symptoms [2]. However, treatment with BoNT does not incur responses in all dystonia patients [3]. For generalized dystonia patients, pharmacotherapies are very limited with higher occurrences of drug-induced side effects [4]. DBS surgery is now approved by CE mark in Europe and by the Food and Drug Administration (FDA) under a humanitarian device exemption (HDE) in the US [4,5]. In 1950, Hess and Hassler performed the first brain stimulation experiments in globus pallidus interna (GPi) on animal models to identify the responses on muscles contractions and postures. Based on their studies, they demonstrated that electrical stimulation in the GPi showed better responses for controlling body postures than stimulation in the thalamic regions and various basal ganglia structures [6,7,8]. In the late 1990s, DBS surgery became very popular after Professor Alim Louis Benabid’s discovery of subthalamic nucleus (STN) DBS for targeting of PD [9,10]. At the same time, pallidal DBS also became popular for dystonia, and the GPi became the area of interest for targeting in DBS surgery [11,12,13]. A few years later, Sanghera et al. compared his hypothesis in their study with 15 patients with dystonia and 78 patients with PD [14,15,16]. They reported that GPe and GPi neurons displayed similar discharge rates and discharge patterns in dystonia to that of PD, but firing neurons were significantly lower than those in PD patients. Although most of the patients in their study were under general anesthesia, it overall appeared that discharge rates of GPi neurons were not affected. Later on, Hutchison et al. examined GPi neuronal firing under local anesthesia for 7 out of 11 patients in their study and showed that for those 7 patients GPi neuronal firing was similar to other PD patients. They concluded that a hypoactive basal ganglia output is not a consistent feature of dystonia, and that anesthesia may have a marked influence on basal ganglia firing rates and patterns [16,17,18].

In this study, our objective was to evaluate our various dystonia patients’ improvements over 1 to 7 years post-DBS in GPi and highlight the importance of proper DBS programming patterns for long-lasting patient improvements. 

## 2. Methods

### 2.1. Study Subjects

We analyzed a total of 67 patients with dystonia aged between 38 to 78 years coming from four centers (India, Kuwait, Egypt, and Turkey). Our patient recruitment numbers (from January 2014 to December 2020) are shown in Table 1. Table 2 presents the different diagnoses according to the MDS dystonia rating scale before DBS. Table 3 illustrates the quality of life of these patients before DBS.

This study was designed to analyze the long-term efficacy of DBS surgery in dystonia patients and was approved by all institutional ethical committees. 

### 2.2. Global Dystonia Severity Scale (GDS) and Burke–Fahn–Marsden Dystonia Rating Scale (BFMDRS) Evaluation

The motor responses to the DBS surgery were retrospectively measured for each patient using the GDS (total score 140) and the BFMDRS scale (total score 120); this was done before DBS surgery and 1, 3, 6, 12, 24, 36, 48, 60, 72, and 84 months post-DBS surgery during follow-up visits. We presented individual patient GDS and BFMDRS scoring as well as the *p* value. 

### 2.3. Neuropsychological and Psychiatric Evaluations

For all patients (before DBS surgery) neuropsychological evaluations were performed using the Kutcher generalized social anxiety scale (KGSAS) and the Warwick– Edinburgh Mental Well-being Scale (WEMWBS), Table 4.

### 2.4. Operative Technique

**Figure 1:**
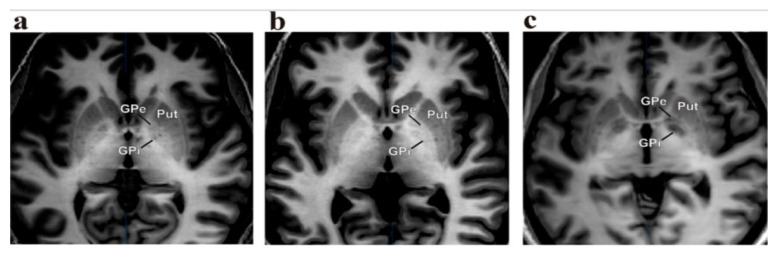
(**a**–**c**) A high-resolution T1-weighted, inversion recovery (IR)-weighted contrast-enhanced MRI used to obtain better clarity of the GPi nucleus with GPe and putamen.

**Figure 2:**
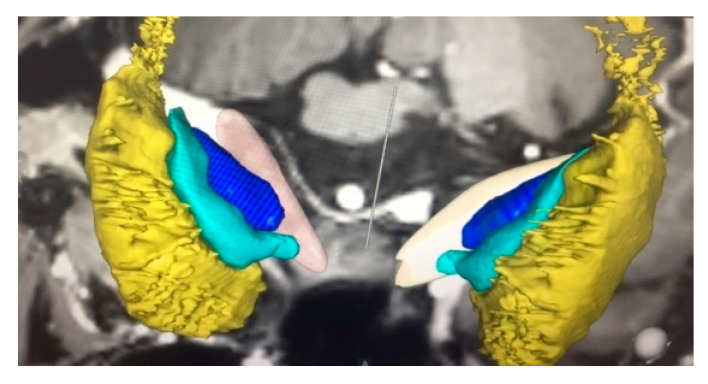
Graphical representation of GPi, GPe, and Putamen. Yellow portion refers Putamen, light green portion refers GPe, and dark blue portion refers GPi nucleus. (This graphical diagram was made by using Medtronic sure tune software.)

The target for dystonia is located in the posteroventral lateral GPi, and it is the same that has been used for PD. This targeting is slightly anterior to the usual pallidotomy target to avoid spreading current to the internal capsule, as occurs with higher amplitudes of stimulation, which is very important in dystonia programming [14,15]. The target is usually chosen 20 mm to 22 mm lateral to and 4 mm below the intercommissural line and 2–3 mm anterior to the intercommissural midpoint. However, various third ventricle shapes and sizes can provide misleading information in establishing a reference point as the “midline”. In this context, it is better to consider the “laterality” of the target as 18 mm lateral to the border of the lateral ventricle [16,17]. MRI brain images with IR sequences are crucial to determine the brain anatomy with the targeted nucleus’ (GPi or STN) proper condition; stereotactic imaging systems are necessary to identify the proper nucleus and perform the planning for the surgery [16,17]. In our centers, we used either the Leksell^®^ frame (Elekta Inc., Stockholm, Sweden) or Cosman-Roberts-Well’s frame (CRW; Integra Radionics, Burlington, MA, USA), where the ring is mounted on the head of the patient and a stereotactic computerized tomography (CT) was performed without contrast using a slice thickness of 1 mm. Then, the MRI and CT images were merged, and stereotactic coordinates were obtained with the Frame link 5 software on a Stealth Station (Medtronic, Minneapolis, MN, USA). A high-resolution T1-weighted, inversion recovery (IR)-weighted (slice thickness 2 mm without gap) and contrast-enhanced MRI was usually obtained for better clarity of the GPi nucleus (Figure 1 and Figure 2).

Intraoperative macro stimulation test through the microelectrode was first performed at 1.0–4.0 V, 200 µs, and 130 Hz using trajectories and depths that revealed pallidal firing patterns, to check responses at the level of the target and below the target to find the threshold for internal capsule response. MER provides important real-time information that other localization techniques simply do not [19,20,21,22]. In GPi targeting for dystonia, extrinsic responses can come from optic tract stimulation, which results in phosphenes or the perception of flashes, and from internal capsule stimulation, which results in tonic contraction of the contralateral face or extremities [23,24,25,26]. In intrinsic responses, patients feel tightness or paresthesia. Alternatively, this same surgery is usually performed under general anesthesia for pediatric patients as well as in adults for those who have severe dystonic postures. In dystonia cases, bilateral GPi lead placement can also be done under local anesthesia or general anesthesia depending on the patient’s condition and posture [27,28,29,30]. 

### 2.5. Post-Operative DBS Programming

The improvement of dystonia in pallidal stimulation could be delayed, and it can take several months to get a response. In our experience, phasic dystonic movements respond early to stimulation, whereas tonic components take several weeks to respond well in post-surgery stimulation. All patients had a preoperative brain MRI to calculate the electrode trajectory and target. Post-operatively, the correct electrode positioning was confirmed by computer tomography in all patients and evaluated by the operating neurosurgeon in each center. As long as the initial programming settings are involved, two different patterns were applied. One group of patients (32 patients) initially had bipolar configurations, using the 1st most ventral contact as negative and 2nd most ventral contact as positive with 210 microseconds (µs) pulse width and 130 Hz frequency. The amplitude initially was kept between 2.0 V to 3.0 V depending on the patient’s responses post-surgery before discharge. The second group of patients (35 patients) had a monopolar configuration pattern of programming where IPG was kept as positive and the 2nd most ventral contact was kept negative with pulse width of 90 microseconds (µs), frequency 150 Hz, and amplitude varied between 2 and 4 V. In both groups of patients, stimulation parameters gradually increased over a period of 3 months after hospital discharge. 

### 2.6. Statistical Analysis

Due to non-normal data distribution, a Mann–Whitney U test was used to compare continuous independent factors including generator parameter values, GDS, and BFMDRS scores between groups. Spearman correlation coefficient was used for correlation analyses between generator life and generator pulses. Bonferroni correction was used to account for multiple comparisons. *p* < 0.05 was considered statistically significant. The statistical analyses were done using SPSS versions 24.0 (SPSS Inc., Chicago, IL, USA).

## 3. Results

### 3.1. Post-Operative 2014 Batch of 10 Patients’ Improvement (3 Months to 84 Months Post-DBS)

**Figure 3:**
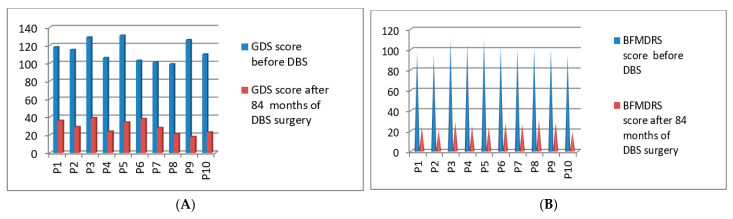
GDS (**A**) and BFMDRS (**B**) scores before DBS and after 84 months of DBS surgery.

Post-operatively, we started the clinical evaluations systematically using GDS and BFMDRS scores for the first 10 patients who underwent DBS surgery in the year of 2014. Significant improvement was seen between the preoperative state and 84 months post-stimulation. The GDS *p*-value was 0.017 after 84 months post-DBS stimulation, which showed improvement of more than 86% for those patients, and the BFMDRS *p*-value was 0.022 after 84 months. Detailed GDS and BFMDRS scorings were calculated and described in Figure 3A,B. Full details of GDS and BFMDRS scoring are shown in Table 5.

### 3.2. Post-Operative 2015 Batch of 9 Patients’ Improvement (3 Months to 72 Months Post-DBS)

**Figure 4:**
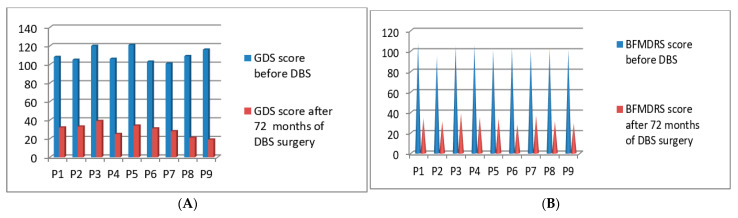
GDS (**A**) and BFMDRS (**B**) scores before DBS and after 72 months of DBS surgery.

Post-operatively we started the clinical evaluations systematically using GDS and BFMDRS scores for the second batch of 9 patients who underwent DBS surgery in the year of 2015. The GDS *p*-value was 0.021 after 72 months and the BFMDRS *p*-value was 0.028 after 72 months post-DBS stimulation (Figure 4A,B). Full details of GDS and BFMDRS scoring are shown in Table 5.

### 3.3. Post-Operative 2016 Batch of 11 Patients’ Improvement (3 Months to 60 Months Post-DBS)

**Figure 5:**
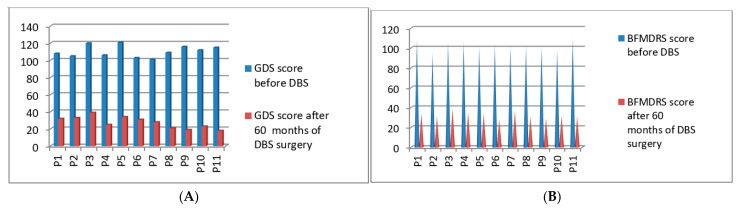
GDS (**A**) and BFMDRS (**B**) scores before DBS and after 60 months of DBS surgery.

Post-operatively, we started the clinical evaluations systematically using GDS and BFMDRS scores for the third batch of 11 patients who underwent DBS surgery in the year of 2016. The GDS *p*-value was 0.021 after 60 months and the BFMDRS *p*-value was 0.029 after 60 months post-DBS stimulation (Figure 5A,B). Full details of GDS and BFMDRS scoring are shown in Table 5. 

### 3.4. Post-Operative 2017 Batch of 7 Patients’ Improvement (3 Months to 48 Months Post-DBS)

**Figure 6:**
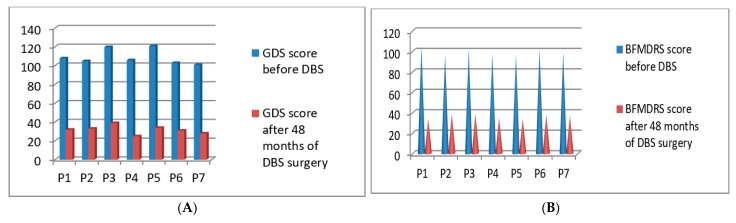
GDS (**A**) and BFMDRS (**B**) scores before DBS and after 48 months of DBS surgery.

Post-operatively we started the clinical evaluations systematically using GDS and BFMDRS scores for the fourth batch of 7 patients who underwent DBS surgery in the year of 2017. The GDS *p*-value was 0.039 after 48 months and the BFMDRS *p*-value was 0.044 after 48 months post-DBS stimulation (Figure 6A,B). Full details of GDS and BFMDRS scoring are shown in Table 5. 

### 3.5. Post-Operative 2018 Batch of 9 Patients’ Improvement (3 Months to 36 Months Post-DBS)

**Figure 7:**
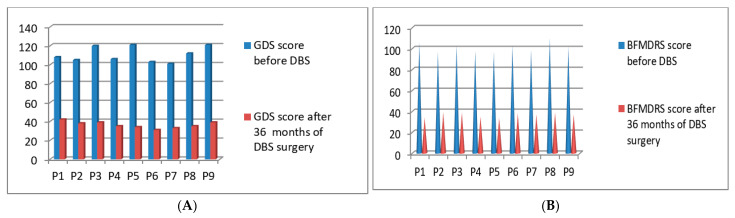
GDS (**A**) and BFMDRS (**B**) scores before DBS and after 36 months of DBS surgery.

Post-operatively we started the clinical evaluations systematically using GDS and BFMDRS scores for the fifth batch of 9 patients who underwent DBS surgery in the year of 2018. The GDS *p*-value was 0.042 after 36 months and the BFMDRS *p*-value was 0.042 after 36 months post-DBS stimulation (Figure 7A,B). Full details of GDS and BFMDRS scoring showed in Table 5. 

### 3.6. Post-Operative 2019 Batch Total 12 Patients’ Improvement (3 Months to 24 Months Post-DBS)

**Figure 8:**
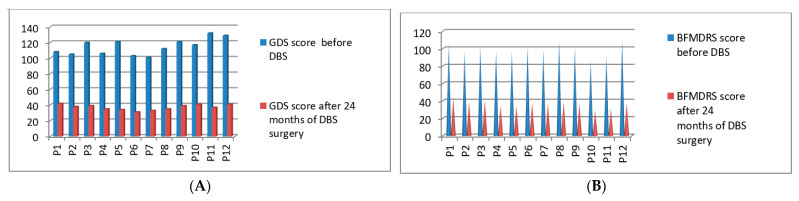
GDS (**A**) and BFMDRS (**B**) scores before DBS and after 24 months of DBS surgery.

Post-operatively, we started the clinical evaluations systematically using GDS and BFMDRS scores for the sixth batch of 12 patients who underwent DBS surgery in the year of 2019. The GDS *p*-value was 0.044 after 24 months and the BFMDRS *p*-value was 0.046 after 24 months post-DBS stimulation (Figure 8A,B). Full details of GDS and BFMDRS scoring are shown in Table 5. 

### 3.7. Post-Operative 2020 Batch of 9 Patients’ Improvement (3 Months to 16 Months Post-DBS)

**Figure 9:**
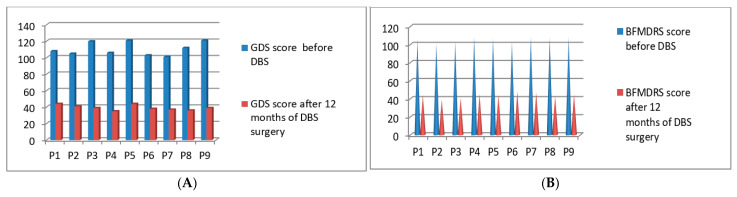
GDS (**A**) and BFMDRS (**B**) scores before DBS and after 12 months of DBS surgery.

Post-operatively, we started the clinical evaluations systematically using GDS and BFMDRS scores for the seventh batch of 9 patients who underwent DBS surgery in the year of 2020. The GDS *p*-value was 0.049 after 16 months and the BFMDRS *p*-value was 0.050 after 16 months post-DBS stimulation (Figure 9A,B). Full details of GDS and BFMDRS scoring showed in Table 5. 

## 4. Discussion

DBS has been an effective and worldwide established therapy for decades. Many articles have been published regarding the importance of DBS surgery for PD patients with demonstrated significant improvements [31,32,33]. However, DBS for focal, segmental, and generalized dystonia is not used as often. Few articles previously demonstrated the utility of DBS for dystonia, but the reported clinical improvement was less than what we have observed. The meta-analysis by Moro et al. showed an average BFMDRS score improvement of more than 65%, depending on the patient´s severity [34,35,36,37,38].

In our study, the mean BFMDRS and GDS improvement was nearly 56 ± 1.0 and 68 ± 1.0 after DBS surgery after 6 to 12 months, respectively, with the majority of patients diagnosed with generalized and cervical dystonia. However, this study majorly demonstrated that in patients with longer stimulation (more than 5 years to 7 years) the improvement was much better, and GDS and BFMDRS scores of 30 ± 1.0 and 26 ± 1.0, respectively, can be achieved. So far, no studies showed such an improvement in patients with dystonia post-DBS stimulation. None of the patients became bed-ridden post-stimulation. Some patients showed very slow improvement post-DBS. Forty out of 67 patients became more social, and 12 young patients went back to work. Five patients with depression post-DBS were continuously monitored by their psychiatrists with close medication control. Two out of 67 patients came for frequent programming settings as those patients initially showed severe dysarthria as adverse effects of stimulation. Five out of 67 patients came with post-surgery infection requiring bilateral leads revision surgery once again, and after lead revision surgery they showed consistently good improvements. Post-operatively, in 1 patient who was earlier diagnosed with depressive behavior with suicidal tendency, the symptoms worsened after the first programming and subsided after the second programming. Otherwise, 66 out of 67 patients showed continuous improvement post-surgery.

Compared to all dystonia groups, the post-stroke hemi-dystonia patients showed the maximum improvement with 75% reduction in symptoms 3 weeks post-surgery. After 3 months, these patients showed 80% improvement in symptoms with initial stimulation itself; at 6 months post-surgery, those patients that underwent second programming for higher stimulation changes showed a 90% control of the symptoms. Improvement continued after 12 months post-surgery, and follow-up visits continued. The preoperative mean GDS was 10.79 ± 1.0 for generalized dystonia and 11.79 ± 1.0 for cervical dystonia patients. 

All patients had Medtronic quadripolar 3387 DBS electrode lead placements (Medtronic, Minneapolis, MN, USA), which have 1.5 mm gaps between the single contacts in both sides of the GPi. Forty-seven patients had Medtronic Activa RC implanted pulse generator (IPG), and 20 patients had Medtronic Activa PC IPG implanted. Twenty patients chose non-rechargeable IPGs for cost savings, and 45 patients chose rechargeable IPGs to avoid future replacement surgeries. The most common stimulation-related side effects were dysarthria, impaired upper and lower limb coordination, and impaired balance. These were transient and subsided after modifying programming settings. Stimulation variables, including the active contact(s), amplitude in volts, pulse width (PW) in microseconds, and frequency in hertz (Hz) and indicators of implantable pulse generator (IPG) longevity (impedance, current drain, and battery voltage) were recorded at each follow up visit. According to the existing literature, it has been suggested that the earlier the surgical intervention for dystonia, the better the outcome [39,40,41,42]. However, regarding this hypothesis, controversial results have also been published [42,43,44]. 

In our study, the duration of the disease or age did not correlate with the clinical responses of post-DBS patients. Interestingly, five patients with post-stroke hemi-dystonia had very good response, despite the fact that post-stroke DBS is considered controversial. In our study, a few patients with younger ages returned to their profession after DBS relative to older patients at the time of surgery. Our study showed no fatal side effects related to surgery or post-stimulation. Our adverse effects were not more than severe than other studies that were published before. We used various frequencies and pulse widths in programming our patients, starting at a very slow pace with each step monitored closely by neurologists. We used high pulse widths for a few patients, but we kept the amplitude on the lower side to overcome stimulation-induced side effects. Although we have observed similar post-stimulation-induced side effects as seen by Moro et al. in their study, in our study adverse effects were not as severe. The stimulation-related coordination in upper and lower limbs indicated most probably involvement of the internal capsule, which resolved after the second programming [44]. Tagliati et al., 2011, observed that the proportion of stimulation-related speech impairment was quite high at more than 35% [24]. We have seen two patients with post-stimulation speech impairments where dystonic posture was corrected in more than 45%. The results of speech impairment suggested posteriorly located contact activation. When we changed the contact, speech-related problems resolved within 5–10 min. It remains a question to see if a directional lead can completely overcome current spreading posteriorly and related speech issues. Few earlier studies described utilization of higher stimulation parameters and probably stimulating more GPe area than GPi induced Parkinsonian symptoms in patients [44]. 

In our study we observed that 2 out of 67 dystonia patients developed severe post-DBS freezing of gait at higher amplitude, but after modifying the programming parameters all parkinsonian symptoms resolved immediately. Psychiatric or cognitive adverse events were rare, but in our study five patients had depression pre- and post-surgery. Few articles also demonstrated earlier IPG-related complications associated with infections, leads fractures, and hardware-related issues. However, in this study we did not see any complications associated with IPGs hardware or lead fractures.

## 5. Conclusions

This study demonstrates DBS as an effective treatment option for patients with different dystonia types such as generalized dystonia, cervical dystonia, and various brain pathology induced dystonias. This treatment is usually safe and well-tolerated. This study primarily demonstrates that an extensive stimulation period always gives better responses for dystonia patients. Post-DBS surgery infections can occur, so there is a need for precautions and proper hygiene maintenance after DBS surgery. Post-stimulation adverse events may occur but can be avoided if intensive programming is made as early as possible. Thus, we feel that more emphasis should be put upon increasing awareness of this DBS treatment option in severe drug-resistant dystonia patients. 

## Figures and Tables

**Table 1:** Total numbers of patients that underwent bilateral GPi DBS surgery for dystonia at four centers.

Year of the Surgery	Total Number of Patients	Placements of the Electrodes
January 2014 to December 2014	10 (7 males and 3 females)	Bilateral GPi
January 2015 to December 2015	9 (5 males and 4 females)	Bilateral GPi
January 2016 to December 2016	11 (6 males and 5 females)	Bilateral GPi
January 2017 to December 2017	7 (2 males and 5 females)	Bilateral GPi
January 2018-December 2018	9 (7 males and 2 females)	Bilateral GPi
January 2019 to December 2019	12 (10 males and 2 females)	Bilateral GPi
January 2020 to December 2020	9 (5 males and 4 females)	Bilateral GPi

**Table 2:** Diagnosis of the dystonia patients according to the MDS GDS scaling.

Diagnosis	Total Number of Patients
Generalized Dystonia	30 (20 patients DYT-1 positive and 10 patients DYT-3)
CERVICAL DYSTONIA	27 (17 patients DYT-5 positive and 10 patients DYT-6)
Blepharospasm with PISA syndrome associated with Parkinson’s disease	5
Post-stroke Hemi Dystonia	5

**Table 3:** Quality of life of dystonia patients before DBS surgery.

Quality of Life with Dystonia	Total Number of Patients with Dystonia
Completely dependent and wheelchair bound	20
Partially able to do social things with dependency	30
Completely independent with dystonia	17

**Table 4:** Neuropsychological assessments before DBS surgery.

Total Number of Patients	Neuropsychological Assessment	Scale Performed
11	Severe depression with total social withdrawal	WEMWBS and KGSAS
4	Moderate depression with suicidal tendency	WEMWBS and KGSAS
1	Severe depression with suicidal tendency	WEMWBS and KGSAS
51	Minimal depression associated with dystonia	WEMWBS and KGSAS

**Table 5:** Full details of GDS and BFMDRS scoring before and after DBS.

Patients No. (Total Patients No. = 67)	Year DBS Undergone	Sex	Age	Dystonia Types	GDS Base Score before DBS	BFMDRS Base Score before DBS	GDS Base Score after DBS	BFMDRS Base Score after DBS	Post DBS Total Timeline (GDS and BFMDRS Scaling Done Post DBS)
P1	2014	M	44	Generalized	118/140	98/140	36/140	24/120	84 months
P2	2014	M	56	Generalized	115/140	95/120	29/140	21/120	84 months
P3	2014	F	48	Generalized	129/140	110/120	39/140	29/120	84 months
P4	2014	M	39	Generalized	106/140	106/120	24/140	25/120	84 months
P5	2014	M	53	Generalized	131/140	111/120	34/140	24/120	84 months
P6	2014	F	65	Generalized	103/140	103/120	38/140	28/120	84 months
P7	2014	F	55	Cervical	101/140	101/120	28/140	27/120	84 months
P8	2014	M	67	Cervical	99/140	102/120	21/140	31/120	84 months
P9	2014	M	78	Cervical	126/140	101/120	18/140	29/120	84 months
P10	2014	M	70	Cervical	110/140	95/120	23/140	19/120	84 months
P1	2015	M	68	Generalized	108/140	108/120	32/140	34/120	72 months
P2	2015	F	55	Generalized	105/140	95/120	33/140	31/120	72 months
P3	2015	F	48	Generalized	120/140	105/120	39/140	39/120	72 months
P4	2015	M	38	Generalized	106/140	106/120	25/140	35/120	72 months
P5	2015	M	41	Cervical	121/140	101/120	34/140	34/120	72 months
P6	2015	M	70	Cervical	103/140	103/120	31/140	28/120	72 months
P7	2015	F	59	Cervical	101/140	101/120	28/140	37/120	72 months
P8	2015	F	61	Cervical	109/140	102/120	21/140	31/120	72 months
P9	2015	M	40	Generalized	116/140	101/120	19/140	29/120	72 months
P1	2016	M	70	Generalized	108/140	108/120	32/140	34/120	60 months
P2	2016	M	67	Post Stroke Hemi dystonia	105/140	95/120	33/140	31/120	60 months
P3	2016	M	53	Generalized	120/140	105/120	39/140	39/120	60 months
P4	2016	F	46	Generalized	106/140	106/120	25/140	35/120	60 months
P5	2016	M	58	Generalized	121/140	101/120	34/140	34/120	60 months
P6	2016	F	39	Blepharospasm With PISA syndrome	103/140	103/120	31/140	28/120	60 months
P7	2016	F	48	Cervical	101/140	101/120	28/140	37/120	60 months
P8	2016	M	50	Cervical	109/140	102/120	21/140	31/120	60 months
P9	2016	F	51	Generalized	116/140	101/120	19/140	29/120	60 months
P10	2016	F	65	Generalized	112/140	98/120	23/140	32/120	60 months
P11	2016	M	42	Generalized	115/140	110/120	18/140	31/120	60 months
P1	2017	M	51	Generalized	108/140	106/120	32/140	34/120	48 months
P2	2017	M	48	Generalized	105/140	97/120	33/140	39/120	48 months
P3	2017	F	53	Generalized	120/140	103/120	39/140	39/120	48 months
P4	2017	F	43	Cervical	106/140	98/120	25/140	35/120	48 months
P5	2017	F	45	Blepharospasm With PISA syndrome	121/140	97/120	34/140	34/120	48 months
P6	2017	F	65	Cervical	103/140	103/120	31/140	38/120	48 months
P7	2017	F	66	Cervical	101/140	99/120	28/140	37/120	48 months
P1	2018	F	51	Generalized	108/140	106/120	42/140	34/120	36 months
P2	2018	M	56	Generalized	105/140	97/120	38/140	39/120	36 months
P3	2018	M	41	Generalized	120/140	103/120	39/140	39/120	36 months
P4	2018	M	42	Post Stroke Hemi dystonia	106/140	98/120	35/140	35/120	36 months
P5	2018	F	63	Cervical	121/140	97/120	34/140	34/120	36 months
P6	2018	M	72	Cervical	103/140	103/120	31/140	38/120	36 months
P7	2018	M	63	Post Stroke Hemi dystonia	101/140	99/120	33/140	37/120	36 months
P8	2018	M	59	Cervical	112/140	110/120	35/140	39/120	36 months
P9	2018	M	71	Generalized	121/140	102/120	39/140	37/120	36 months
P1	2019	M	60	Generalized	108/140	106/120	42/140	44/120	24 months
P2	2019	M	61	Generalized	105/140	97/120	38/140	39/120	24 months
P3	2019	M	52	Cervical	120/140	103/120	39/140	41/120	24 months
P4	2019	M	43	Cervical	106/140	98/120	35/140	35/120	24 months
P5	2019	F	41	Cervical	121/140	97/120	34/140	34/120	24 months
P6	2019	M	48	Blepharospasm With PISA syndrome	103/140	103/120	31/140	38/120	24 months
P7	2019	M	63	Cervical	101/140	99/120	33/140	37/120	24 months
P8	2019	F	56	Cervical	112/140	110/120	35/140	39/120	24 months
P9	2019	M	55	Post Stroke Hemi dystonia	121/140	102/120	39/140	37/120	24 months
P10	2019	M	45	Generalized	117/140	86/120	41/140	29/120	24 months
P11	2019	M	39	Cervical	132/140	92/120	37/140	31/120	24 months
P12	2019	M	58	Generalized	129/140	110/120	41/140	38/120	24 months
P1	2020	M	38	Cervical	108/140	106/120	44/140	44/120	12 months
P2	2020	F	45	Cervical	105/140	101/120	41/140	39/120	12 months
P3	2020	M	52	Post Stroke Hemi dystonia	120/140	103/120	39/140	41/120	12 months
P4	2020	M	54	Cervical	106/140	108/120	35/140	45/120	12 months
P5	2020	M	65	Blepharospasm with PISA syndrome	121/140	107/120	44/140	44/120	12 months
P6	2020	F	75	Cervical	103/140	103/120	38/140	48/120	12 months
P7	2020	M	71	Cervical	101/140	109/120	37/140	47/120	12 months
P8	2020	F	69	Blepharospasm With PISA syndrome	112/140	108/120	36/140	43/120	12 months
P9	2020	F	62	Generalized	121/140	108/120	39/140	45/120	12 months

## Data Availability

All data generated or analyzed during this study are included in this article. Further enquires can be directed to the corresponding author.
